# Modulation of Detoxification, Immune, and Epigenetic Systems by Two Aryl Organophosphorus Flame Retardants During Early Development in Zebrafish

**DOI:** 10.3390/toxics13090794

**Published:** 2025-09-18

**Authors:** Montserrat Solé, Sílvia Joly, Sergi Omedes, Isabel Forner-Piquer, Laia Ribas

**Affiliations:** Institut de Ciències del Mar, Consejo Superior de Investigaciones Científicas (ICM-CSIC), 08003 Barcelona, Spain; msole@icm.csic.es (M.S.);

**Keywords:** organophosphorus flame retardants, carboxylesterases, epigenetics, immune system, detoxification

## Abstract

Organophosphorus flame retardants (OPFRs) are emerging alternatives to halogenated compounds, yet their environmental toxicity remains underexplored. This study evaluated the developmental toxicity of two aryl-OPFRs, triphenyl phosphate (TPP) and tricresyl phosphate (TCP), in zebrafish (*Danio rerio*) from 2 h to 5 days post fertilization (hpf–dpf). Survival, hatching rate, and malformations were assessed across concentrations of 250–1000 µg/L, alongside with gene expression analysis at 5 dpf (250 and 500 µg/L) targeting detoxification (*ces2*), immune responses (*il1β*, *casp9*), and epigenetic markers (*dnmt1*, *dnmt3*). In vitro enzymatic assays evaluated interactions of both aryl-OPFRs with carboxylesterase (CE) and acetylcholinesterase (AChE) enzymes. While no significant morphological effects were observed, TPP showed higher toxicity than TCP. Notably, TCP (500 µg/L) downregulated genes linked to metabolism and immunity. CE activity and *ces2* modulation may suggest CE as a potential biomarker for aryl-OPFR exposure. These findings, although at concentrations above the environmental ones, may be valuable for mechanistic purposes and underscore the need for further investigation in developmental toxicity given their lipophilic nature and distinct molecular responses.

## 1. Introduction

Flame retardants are regarded as plastic additives aimed to confer lower inflammability properties to industrial and commercial products [1]. In the market, the formerly employed polybrominated diphenyl ethers (PBDEs) have been progressively replaced by non-halogenated flame retardants with presumably lower biological reactivity and toxicity [2]. Among the newly introduced replacements, organophosphate flame retardants (OPFRs) stand out. They comprise a large group of diverse substances with a structural similarity to organophosphorus (OP) pesticides [3]. Given this resemblance, it is not unreasonable to think that they may similarly interact with biological molecules. Because of their lipophilic nature and widespread applications, OPFRs have been detected in different matrices, as well as wild fauna, human blood, or aquatic environments at concentrations mostly at the ng/L level but occasionally reaching µg/L [4,5,6,7,8].

Triphenyl phosphate (TPP) and tricresyl phosphate (TCP) are two aryl-OPFRs that interact with the carboxylesterases (CEs) family by binding to the active site of the enzyme, following a similar mechanistic phosphorylation than the OP pesticides [9]. Indeed, the European Chemicals Agency (ECHA) added the TPP in the candidate list for “Substance of very high concern” because of its endocrine-disrupting properties.

CEs are a family of enzymes from the α/β hydrolases family involved in the hydrolysis of a large variety of ester and amide compounds [10]. It is well known that many environmental chemicals interact with these enzymes, potentially preventing their catalytic role. They are also seen as suicidal enzymes preventing the inhibition of the neurotransmitter enzyme acetylcholinesterase (AChE) after OP exposures [11,12].

The innate immune response is the earliest immune mechanism. It is characterized by its nonspecific nature and does not rely on prior recognition of an invader’s surface structures [13]. This response is advantageous as it can be triggered by external molecules, and it is always present, reacting quickly through inflammatory pathways [14]. Interleukins play a crucial role in immune defense by recruiting leukocytes to infection sites to initiate inflammation. Interleukin 1 beta (IL1β) is a key player in the initial inflammatory response [15]. Once cleaved by caspase (CASP9) into its mature form, IL1β binds to its receptor, activating the nuclear factor NF-kβ pathway [16]. Caspases are essential for apoptotic pathways and the cell’s inflammasome [17]. In particular, CASP9 is a crucial enzyme in the mitochondria-mediated cell death pathway [18]. OPFRs, as recently reviewed [19], activate the fish immune system, specifically modifying the TLR4/NF-kβ signaling pathway in zebrafish (*Danio rerio*) larvae [20].

DNA methylation, an epigenetic event, is performed by DNA methyltransferases (DNMTs) [21]. In mammals, the primary DNMTs include DNMT1, which is responsible for maintaining DNA methylation, and DNMT3A and DNMT3B, which are responsible for *de novo* DNA methylation [22]. Environmental pollutants can alter DNA methylation patterns by disrupting *dnmt* gene expression during zebrafish embryogenesis and adulthood [23,24,25,26].

Zebrafish is an excellent animal model in many research fields offering several advantages such as the rapid embryonic development within 72 h post fertilization (hpf), progressing into the larval stage by 5 days post fertilization (dpf) [27,28]. Over the past two decades, zebrafish has been extensively used as a model in aquatic toxicology [29] and adopted as a primary test species by the Organization for Economic Cooperation and Development [30].

It is known that aryl-OPFRs can be bioaccumulated in aquatic species [8] and have been reported to interfere with several pathways in zebrafish [31,32,33,34,35]. Here, we exposed zebrafish embryos to different concentrations of TPP and TCP during early development. We assessed whether this short-term exposure affects development, detoxification, immune, and epigenetic mechanisms. It included the expression of key genes involved in detoxification (*ces2*), innate immune response (*il1β* and *casp9*), and epigenetics (*dnmt1* and *dmnt3b2*, further referred to as *dnmt3*) in an in vivo fish model species. We also performed enzymatic in vitro interactions of TPP and TCP with two B-esterases (CE and AChE) in whole zebrafish tissue homogenates. By using non-environmental concentrations of both compounds, our study adopted mechanistic purposes rather than resemble real-life scenarios. The consequences of the diverse, or lack thereof, effects that high concentrations of OPFRs may cause on early-life zebrafish development are further discussed.

## 2. Materials and Methods

### 2.1. Flame Retardant Solutions

Triphenyl phosphate (TPP; CAS 115-86-6; log K_OW_ = 4.59, C_18_H_15_O_4_P, >99% purity, Sigma-Aldrich, St. Louis, MO, USA) and Tricresyl phosphate (TCP; CAS 1330-78-5; log K_OW_ = 6.34, C_21_H_21_O_4_P, >89% purity, Sigma-Aldrich, St. Louis, MO, USA) were dissolved in dimethyl sulfoxide (DMSO, Sigma-Aldrich, St. Louis, MO, USA) to obtain a stock concentration of 1 mg/mL. Aliquots of the stock concentrations were kept at −20 °C. Stock dilutions were diluted in ultrapure water to obtain the working concentrations (250, 500 and 1000 µg/L) with a maximum concentration of 0.01% DMSO. In addition, a control group with no OPFRs (only ultrapure water, group referred as CTRL) and a solvent control with 0.01% DMSO, group referred as Carrier, were included in the experimental setup. The treatment started at 2 h post fertilization (hpf) until 5 days post fertilization (dpf). All water solutions were renewed daily, and the treatments were performed four consecutive times in different biological pairs, and each time was considered as an experimental replicate (n = 4).

### 2.2. Animal Rearing Conditions

Adult zebrafish (AB wild-type strain, ZFIN ID: ZDB-GENO-960809-7) were kept in the experimental facilities at the Institute of Marine Sciences (ICM-CSIC) in Barcelona (Spain) under standardized water conditions (28 ± 0.2 °C; pH 7.2 ± 0.5 and 12/12 h light/dark photoperiod) in a closed recirculating system equipped with a 3000 L/h water pump and a UV light system [28]. Air temperature of the chamber was set at 26 ± 1 °C and humidity levels were around 60 ± 3%. Daily monitoring ensured appropriate physicochemical conditions, including water temperature, pH, conductivity (750–900 μS), and dissolved oxygen (6.5–7.0 mg/L) [28]. Weekly checks for sulfite, sulfate, nitrate, and ammonia were performed using commercial kits and periodically verified by the ICM-CSIC water analysis service. Adult fish were fed ad libitum twice daily with dry pellets (AquaSchwarz, manufactured in Hohen Neuendorf, Germany) and with live *Artemia nauplii* (AF48, INVE Aquaculture, Dendermonde, Belgium).

Zebrafish embryos were generated by natural spawning from different breeding families to maintain the interfamily variation [36]. The breeding couples were separated the afternoon before into different crossing tanks. Next morning, the tank dividers were removed letting them spawn. Then, the eggs were collected and rinsed with E3 embryo media (pH 7.2 ± 0.5). Dead eggs were removed. The total number of fertilized eggs was counted to ensure fertility matched the reference values for this species, and post-hatch survival rates were consistent with OECD guidelines for the Fish Sexual Development Test [37]. Groups of 50 fertilized eggs were randomly placed in each Petri dish with E3 embryo media supplemented with 0.1% methylene blue (Sigma-Aldrich). Petri dishes were placed inside an incubator at 28 ± 0.5 °C until the start of the experiment.

### 2.3. Experimental In Vivo Design

Embryos at 2 hpf, from the same breeding couple, were individually transferred to 96-well plates. Each multi-well plate was divided in 8 groups with a total of 12 embryos per group: CTRL (ultrapure water, no OPFRs), Carrier (ultrapure water + DMSO at 0.01%), TPP at 250 µg/L, 500 µg/L and 1000 µg/L (TPP 250, TPP500, TPP 1000) and TCP at 250 µg/L, 500 µg/L and 1000 µg/L (TCP 250, TCP500, TCP1000). After adding 250 µL of the chemical solution in each well, plates were covered with a transparent adhesive to avoid evaporation. Embryos were kept inside an incubator (28 °C and 12:12 h light-dark photoperiod) and survival, hatching rate and malformations were daily monitored with a Leica EZ4 Stereo Microscope (Leica Microsystem Ltd., Wetzlar, Germany). Larvae development was also recorded individually for each of the experimental groups. To minimize inter-observer error, malformations analyses were conducted by the blinded researcher in the four independent experimental sets. At 5 dpf, larvae from each condition were euthanized by thermic shock, snap-frozen in dry ice and stored at −80 °C until further analysis.

### 2.4. Expression Analysis

Total RNA was individually extracted from larvae (n = 4–6 per group) in CTRL, carrier, TPP250, TPP500, TCP250 and TCP500. The highest TPP and PCP concentrations (1000 µg/L) were not included due to limited number of surviving larvae. The extraction was performed using Quick-RNA Microprep Kit (Zymo Research, Irvine, CA, USA) according to the manufacturer’s instructions. RNA pellets were suspended in 25 μL DEPC–water and stored at −80 °C. RNA concentration was measured with an ND-1000 spectrophotometer (NanoDrop Technologies, Wilmington, DE, USA), and RNA quality was assessed on a 1% agarose/formaldehyde gel electrophoresis. Following supplier protocols, 100 ng of total RNA from each sample was treated with DNase I, Amplification Grade (Thermo Fisher Scientific Inc., Waltham, MA, USA) and reverse-transcribed to cDNA using the Transcription First Strand cDNA Synthesis kit (Roche) with Random hexamers (Invitrogen, Carlsbad, CA, USA). Negative control reactions were performed for all tested genes. Quantitative Reverse Transcription Polymerase Chain Reaction (RT-qPCR) was performed in a CFX device (BioRad, Hercules, CA, USA) with cDNA previously diluted 1:10 with DNase-free water. The reaction mix consisted of 5 μL of 2X qPCRBIO SYBR Green Mix Lo-ROX (PCR Biosystems Ltd, London, UK), 0.5 μL of each forward and reverse primer, and 2 μL of DNase-free water. RT-qPCR was conducted in technical triplicates for each sample. The thermocycler conditions were initial denaturation at 95 °C for 3 min, followed by 39 cycles of 95 °C for 10 s, the annealing temperature at 60 °C for 30 s, and a melt curve analysis (65–95 °C at 0.5 °C/5 s) to verify the amplification of a single product. The dissociation step, primer efficiency curves, and PCR product (100 to 160 base pare) sequencing confirmed the specificity of each primer pair, with efficiencies ranging from 95 to 106%. Primer sequences were designed using Primer3web v4.1.0. More details are provided in Table 1.

RT-qPCR data were collected by SDS 2.3 and RQ Manager software, and relative quantity (RQ) values were calculated following the 2ΔΔCt method [38,39]. Eukaryotic translation elongation factor 1 alpha 1 (*eef1a1l1)* and ribosomal protein L13a (*rpl113a*) were used as housekeeping genes to normalize the PCR data [40].

### 2.5. Baseline Activities of B-Esterases: Carboxylesterase (CE) and Acetylcholinesterase (AChE)

Unexposed zebrafish pools were used for baseline hydrolysis rates determinations of B-esterases. A pool of 25 larvae at 5 dpf constituted one sample, and 6 pools were made for each replicate (n = 6). Each pool was sonicated for 10 s in 300 µL of buffer phosphate 100 mM pH 7.4 containing 1 mM EDTA. After sonication, the homogenate was centrifuged at 10,000× *g* for 15 min. The p-nitrophenyl acetate (pNPA) and p-nitrophenyl butyrate (pNPB) were the substrates for CE determinations, while the acetylthiocholine iodide (ATC) was used for AChE measurements. The detailed methodology was described elsewhere [41,42]. Briefly, 25 μL of the diluted sample and 200 μL of the reaction mixture for each substrate were added to the microplate wells at 1 mM (pNPA and pNPB) final concentration. For AChE activity determination, 25 μL of diluted sample were incubated with 150 μL of 5,5′-dithio-bis-2-nitrobenzoate (DTNB) for 2 min. Afterwards, 50 μL of ATC (at a final concentration of 1 mM) was added. Changes in sample absorbance (in triplicate) were recorded at 405 nm (CE) and 412 nm (AChE) using a TECAN Infinite 200 microplate reader at 25 °C coupled with Magellan V6.0 data analysis software. Results are reported as nmol/min/mg protein.

### 2.6. In Vitro Inhibition Tests of CE and AChE by Aryl-OPFRs

The residual activities (RA) of CE (using pNPB as substrate) and AChE were assayed for TPP and TCP in 5 dpf larvae homogenates (n = 3 pools made out of the 6 from Section 2.5). Also, recombinant human carboxylesterase hCE1 (ref. E0162, Sigma-Aldrich, St. Louis, MO, USA) and purified AChE from electric eel (CAS 9000-81-4, ref. C2888, Sigma-Aldrich, St. Louis, MO, USA) were included in the test to validate the in vitro protocol [41,42]. For these in vitro tests, 10 µL of the carrier DMSO (1% *v*/*v*) or test compound solutions TCP or TCPP at 100 µM (for purified proteins) or 50 µM (in the case of the zebrafish homogenates) were mixed in a final reaction volume of 100 µL. In each case, the incubations at room temperature lasted 15 min as described elsewhere [41]. We also included, in additional wells, the model inhibitors Bis(4-nitrophenyl) phosphate (BNPP; CAS 645-15-8, Sigma-Aldrich, St. Louis, MO, USA) for CE and 1-5-Bis-(4-allydyimethyl)-ammoniumphenyl)pentan-3-one dibromide (BW284c51; CAS 402-40-4, Sigma-Aldrich, St. Louis, MO, USA) for AChE as positive controls. RA was expressed as percentage of the hydrolysis rate with respect to the carrier controls (100%).

### 2.7. Statistics

Statistical analysis was performed using GraphPad 8.0.2 (GraphPad Software Inc., San Diego, CA, USA). One-way ANOVA followed by Dunnett’s or Tukey’s multiple comparisons test was used when data fulfilled the criteria for a parametric test. Otherwise, non-parametric data was analyzed with Kruskal–Wallis and Dunn’s multiple comparison test. Statistical differences were set at *p* < 0.05. Data are expressed as means ± standard error of the mean (SEM). Data in percentage was transformed to fraction and to arcsin to proceed with ANOVA. For the RT-qPCR results, CTRL and carrier were merged as *t*-tests did not denote significant differences in their fold change (*p* > 0.05).

## 3. Results

### 3.1. Survival Rate

**Figure 1:**
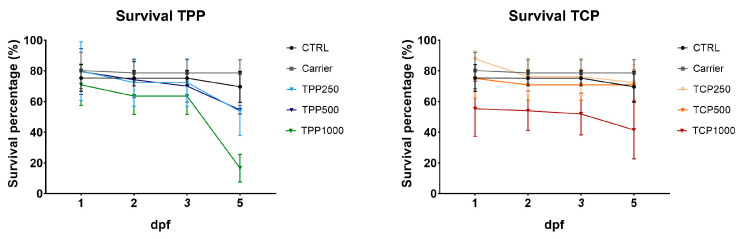
Survival rate of zebrafish embryos/larvae exposed to TCP or TPP. Zebrafish were exposed during early development from 2 h post fertilization (hpf) to 5 days post fertilization (dpf). ANOVA followed by Dunnett’s multiple comparisons test for TPP and TCP at 5 dpf. Data reported as mean ± SEM. Experiments were conducted in quadruplicate with n = 12 individuals/group. No statistical differences were found among treatments when compared to CTRL group at 5 dpf. Abbreviations: tricresyl phosphate (TCP) and triphenyl phosphate (TPP).

No overall statistical differences were found in fish survival for TPP and TCP compared to CTRL at 5 dpf (Figure 1). At 5 dpf, CTRL larvae showed a survival rate of 69.67% similar to the carrier of 78.67%. The groups of TPP resulted in a lower survival percentage of 53.67% (TPP250), 54.67% (TPP500), and 16.67% (TPP1000), while with TCP it was higher 72.42% (TPC250), 70.92% (TPC500), and 41.50% (TPC1000).

### 3.2. Cumulative Hatching Rate

**Figure 2:**
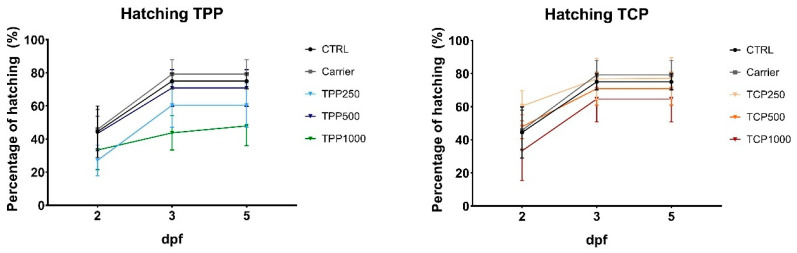
Cumulative hatching rate of zebrafish exposed to TCP or TPP. Zebrafish were exposed during early development from 2 h post fertilization (hpf) to 5 days post fertilization (dpf). Data reported as mean ± SEM. Experiments were conducted in quadruplicate with n = 12 individuals/group. Kruskal–Wallis and Dunn’s multiple comparisons test for TPP and 1-way ANOVA followed by Dunnett’s multiple comparisons test for TCP at 5 dpf. No statistical differences were found among groups. Abbreviations: tricresyl phosphate (TCP) and triphenyl phosphate (TPP).

No differences were found for the hatching rate at 5 dpf for TPP and TCP with respect to the CTRL and carrier groups (Figure 2). At 5 dpf, CTRL larvae showed a hatching ratio of 75% similar to the carrier of 79.17%. The hatching percentage in the exposed groups was 60.42% (TPP250), 70.83% (TPP500), and 47.92% (TPP1000), while in the TCP-exposed group it was slightly higher: 77.08% (TPC250), 70.83% (TPC500), and 64.58% (TPC1000), with no statistical difference among TPP and TCP groups.

### 3.3. Developmental Malformations

**Figure 3:**
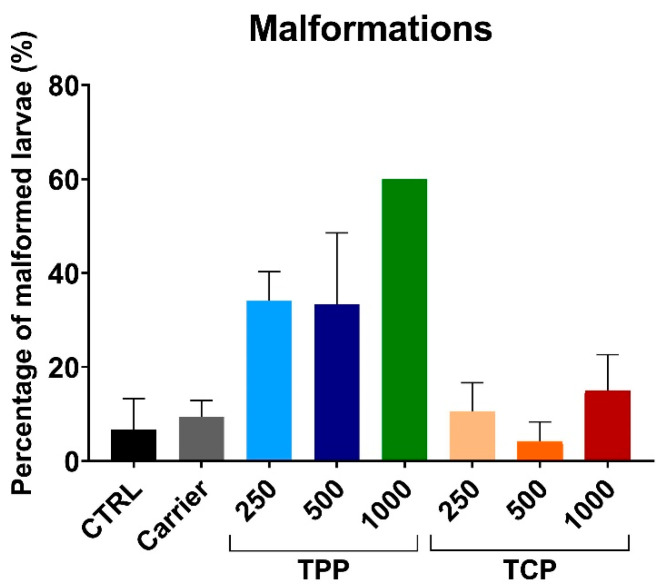
Percentage of malformations observed at 5 dpf zebrafish embryos exposed to different concentrations of TPP and TCP. Data reported as mean ± SEM. Experiments conducted in quadruplicate (n = 12/group). Kruskal–Wallis and Dunn’s multiple comparisons test, no statistical differences were found among groups compared to CTRL or Carrier. Absence of error bar at TPP100 is due to the low survival rate of the embryos; the malformations were assessed in one replicate out of four. Abbreviations: triphenyl phosphate (TPP) and tricresyl phosphate (TCP).

Body malformations were assessed in the eight experimental groups. The signs for malformations included two major types: overall body deformation and tail curvature (Figure S1). The malformations were quantified at 5 dpf (Figure 3). The malformations with the highest concentration of TPP were only scored in one replicate out of four, due to the low survival rate of the larvae. In addition, the TPP500 group presented high variability, ranging from replicates with no malformations to 66.7% of them presenting an abnormal morphology. No statistical differences were found when comparing exposed TPP and TCP groups to CTRL and carrier.

### 3.4. Gene Expression of Detoxification-, Immune-, and Epigenetic-Related Genes

**Figure 4:**
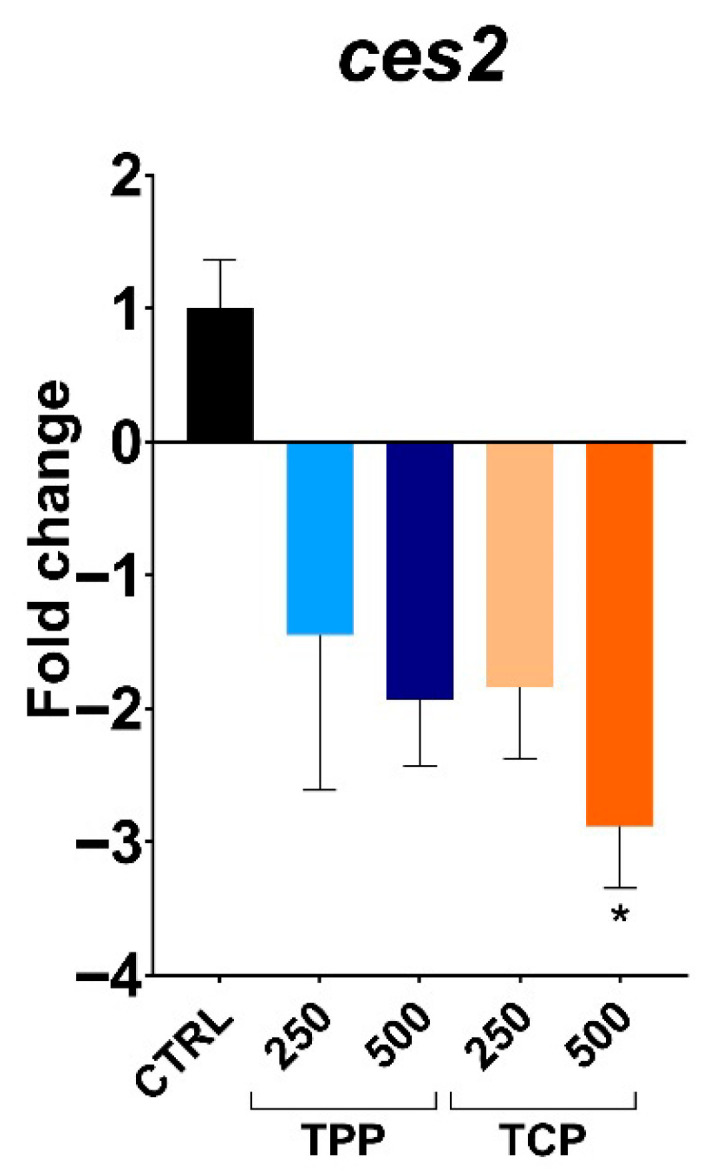
Gene expression profile of carboxylesterase (*ces2*) of 5 days post fertilization (dpf) zebrafish treated with triphenyl phosphate (TPP) and tricresyl phosphate (TCP). Data shown as mean ± SEM. All data were normalized to the expression level of two reference genes (*eef1a1l1* and *rpl113a)* with an arbitrary assigned value of 1. Data refer n = 12 (control), n = 4 (TPP250), n = 5 (TPP500), n = 6 (TCP250), and n = 6 (TCP500) individual larvae. Asterisks indicate significant differences between the treatment and the control group (* *p* < 0.05). Data evaluated by Kruskal–Wallis test followed by Dunn’s multiple comparison test.

Gene expression of *ces2* was downregulated in a concentration-dependent manner with a significant 2.8-fold change decrease at the highest tested concentration of TCP (500 µg/L) compared to the CTRL (Figure 4).

**Figure 5:**
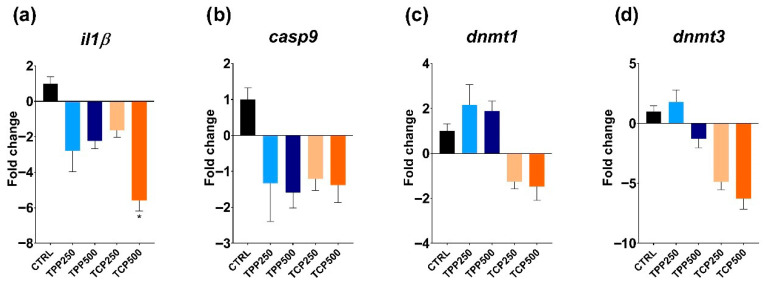
Gene expression profile of 5 days post fertilization zebrafish treated with triphenyl phosphate (TPP) and tricresyl phosphate (TCP): (**a**) *il1b* (* *p* < 0.05), (**b**) *casp9*, (**c**) *dnmt1*, and (**d**) *dnmt3.* Experimental data groups and statistical test as in Figure 4.

Similarly, *il1β* expression was downregulated in all tested groups, with a significant value in the TPC500 group compared to the CTRL (Figure 5a). The expression levels for *casp9* were downregulated but not significantly in any of the tested groups (Figure 5b). For the epigenetic markers, no significant differences were observed for *dnmt1.* However, despite the lack of significance, TPP- and TCP-exposed groups exhibited an opposite response: upregulation for TPP and downregulation for the TCP group (Figure 5c). Interestingly, *dnmt3* transcript levels were clearly downregulated in a concentration-dependent manner for TCP but without reaching statistical significance (Figure 5d).

### 3.5. Basal B-esterase Activities and In Vitro Inhibition by TPP and TCP

**Figure 6:**
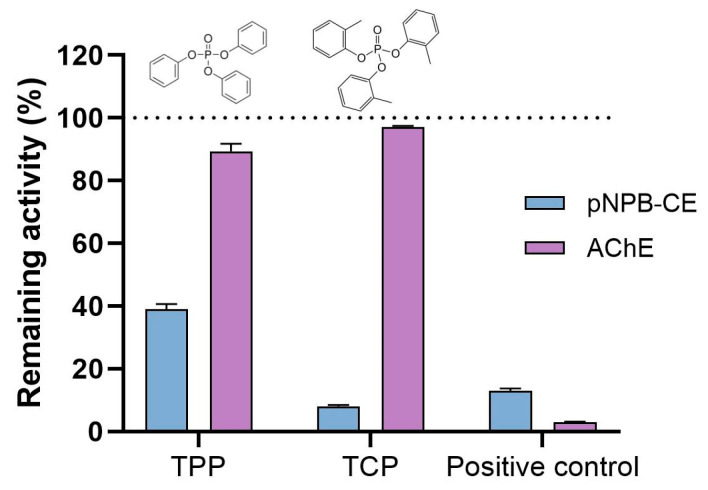
Measurements of carboxylesterase (CE) and acetylcholinesterase (AChE) activities. The remaining activity (expressed in percentage %) was measured as the hydrolysis rate of pNPB (CE) and ATC (AChE) with respect to controls (100%) after incubation of the whole tissue homogenates of 5 dpf zebrafish with TPP and TCP at 50 µM for 15 min. BNPP and BW284c51, at the same concentration, were used as positive controls. Data reported as mean ± SEM; n = 3. All inhibitions over 20% are considered significant [44].

Basal activity of CE was conducted using ρNPA and ρNPB as substrates and ATC for AChE measurements. The hydrolysis rates (nmol/min/mg protein) for the whole tissue homogenates (mean ± SEM; n = 6) were as follows: 87.6 ± 1.8 (ρNPA), 133.6 ± 2.3 (ρNPB), and 849.9 ± 9.6 (ATC). Following a published protocol [43], a single concentration of 100 µM was chosen for testing the interaction with purified proteins (hCE1 and eel AChE), while 50 µM was adopted for the inhibitory potential in 5 dpf zebrafish (Figure 6). At the enzymatic level, the hydrolysis rate of pNPB (adopted for its elevated hydrolysis rate) were TPP (62%), TCP (92%), and BNPP (88%). No inhibition of the basal AChE activity was observed after TPP and TCP incubations, although with the specific AChE inhibitor BW284c51 caused a 97% inhibition (Figure 6).

Likewise, the inhibition reached after the incubation of the two aryl-OPFRs at 100 µM with the purified hCE1 was similar but not for eel-AChE (Table 2).

## 4. Discussion

This study explores (1) the in vivo effects in zebrafish of two commonly used aryl-OPFRs, TPP and TCP, by assessing early development toxicity endpoints and the expression of detoxification-, immune-, and epigenetic-related genes; and (2) the suitability of B-esterases as potential markers of exposure to these flame retardants based on in vitro interference with CE and AChE.

The in vivo results in early developmental stages of zebrafish suggested a higher toxicity in terms of reduced hatching rate, survival, and teratogenic response by exposure to TPP than TCP. Accordingly, lower EC50 and LC50 values for TPP than TCP at 2 and 4 dpf in zebrafish, along with cardiotoxicity and reduced locomotor activity for TPP, have been reported [31]. Decreased swimming speed was also observed in zebrafish embryos exposed to 100 µg/L [35]. The above observations contrast with other findings in which the toxicity attributed to aryl-OPFRs was dose-dependent, and the highest among OPFRs, with TCP affecting the same endpoints (survival, hatching rate, and teratogenicity) but at lower concentrations than TPP [34]. As referred, different concentrations were assayed in the reported studies [31,34] and ours. The highest concentration of 1500 nM (3–1500 nM) [34] contrasts with a maximal 100 µM (0.1–100 µM) [31] and ours (1000 µg/L), equivalent to 3065 nM (TPP; mw = 326.28) and 2700 nM (TCP; mw = 368.36). A lower concentration of TPP in 5 dpf zebrafish (5 µg/L; 15 nM) reduced body length and caused pericardial edema phenotypes [45].

To support the suitability of B-esterases as markers of OPFRs, as pointed out in other animal models [9], basal activities of B-esterases were first characterized in whole tissue homogenates. At the enzymatic level, and considering the whole tissue homogenates, interactions with the hydrolysis rate of ρNPB was higher under in vitro exposure to for TCP (92% inhibition) than to TPP (62%) at 50 µM, and in line with the model OP pesticide BNPP (88%), all at the same 50 µM concentration. However, no inhibitory action by either of these OPFRs was seen on AChE activity, although a 97% inhibition was reached with the model inhibitor BW284c51. An apparent discrepancy between in vivo (neurotoxicity) and in vitro outcome (no AChE inhibition) is not an uncommon feature due to the larger number of factors implicated in the enzymatic and the transcriptomic responses in vivo, as seen with the same two aryl-OPFRs in zebrafish [35].

Likewise, the binding activities of TCP and TPP for AChE showed high affinities [34]; the mechanistic interaction of both aryl-OPFRs with AChE and CE would be expected to be similar, but it was not the case. A particular higher affinity of CEs for aryl-OPFRs could be understood as a protective mechanism preventing AChE inhibition and, therefore, in vivo neurotoxicity. Observations with the crustacean *Daphnia magna* revealed CE was a more suitable marker of neurotoxicity than AChE [46]. Nevertheless, further analysis will be required as differential toxicokinetic or bioaccumulation patterns [47] can likely contribute to the observed in vivo and in vitro outcomes.

The action of TPP and TCP, among other OPFRs, has been thoroughly explored in the early-life stages of zebrafish in terms of general toxicity [31], including transcript modifications, such as in the *ache* gene [34]. Moreover, a dose-dependent inhibition of AChE activity was also observed for TCP, TPP, cresyl diphenyl phosphate (CDP), and the OP pesticide chlorpyrifos [34]. In another study from the same authors, TPP reduced zebrafish AChE activity but not *ache* gene expression, while other neurotransmitters such as γ-aminobutyric acid and histamine and genes involved in larval development were deregulated [35]. In addition, adult zebrafish exposed to TPP exhibited inhibited brain AChE activity and compromised biochemical processes related to the hepatic function [33].

In the present study, in terms of gene expression, a consistent and significant downregulation was recorded at the highest concentration of TCP (500 µg/L) for *ces2* and *il1β* and also for *casp9*, *dnmt1*, and *dnmt3*, although statistical significance was not reached.

Gene expressions for the highest concentrations of TPP and TCP were not assayed due to high toxicity. Nonetheless, a significant downregulation under TCP (500 µg/L) was observed for 2 out of the 5 genes (*ces2* and *il1β*). Although *casp9* and *dnmt3* exhibit a similar pattern, statistical significance was not reached. Our preliminary data pointed towards an inflammatory response; however, a larger sample size would be needed to prove the adequacy of *casp9* as molecular marker. The main immune signaling pathways are conserved in fish, where the innate response serves as the primary defense against immune challenges with a growing importance of adaptive immunity [14,15]. The immune system was activated in larvae and adult zebrafish exposed to the OPFR tris(1-chloro-2-propyl)phosphate (TCPP), altering lipid metabolism in liver and triggering a potential risk of hepatocellular carcinogenesis [48]. Another emerging flame retardant, 2-ethylhexyl diphenyl phosphate (EHDPP), showed that the inflammatory homeostasis by the TLR4/NF-kappa B signaling pathway was altered in zebrafish larvae [20]. In juvenile yellow catfish (*Pelteobagrus fulvidraco*), tris (2-chloroethyl) phosphate (TCEP), another emerging flame retardant, induced apoptosis of fish cells by p53-Bax and caspase-dependent pathways [49]. Nevertheless, scarce data is available regarding the alteration of the immune system by TPP and TCP during early development. Recent data showed that oxidative stress and the immune response were affected in zebrafish gills subjected to TPP (0.1–1 mg/L) for 75 days by increasing the expression of *il13*, *il6*, and complement 3 (*C3*) and *C4*, while the lysozyme activity and the immunoglobulin (IgM) content decreased [50]. In turn, TPP exposure increased oxidative stress at 5 µg/L in zebrafish embryos [45]. Other evidence from mammalian models suggests that OPFRs participate in the modulation of nuclear receptors that encode genes related to detoxification and inflammatory processes [9].

It is recognized that OPFRs are capable of inducing epigenetic changes in fish [51,52,53,54]. Despite an apparent downregulation trend by TCP in the epigenetic markers *dnmt1* and *dmnt3*, like in the case of the immune modulation, a larger sample size would be required to propose these genes as candidate markers of TCP epigenetic modulation. Some studies on zebrafish revealed the toxicity of dioxins (e.g., TCDD) by altering the expression of this particular *dnmts* markers [25]. For example, TCDD exposure during early embryogenesis provoked developmental stage-specific upregulation of *dnmt1* and *dnmt3b2*, coupled with downregulation of *dnmt3a1*, *dnmt3b1*, and *dnmt3b4* [23]. Other data treating developing zebrafish embryos with benzo[a]pyrene, a potent DNA-hypomethylating compound, did not alter either the transcriptional or enzymatic activity of the *dnmts* [55]. Here, we could not confirm a significant alteration of the *dnmt1* and *dnmt3* transcripts, but a downregulation trend for TCP was outlined. As commented before, a larger sample size would have been beneficial to support the role that OPFR might have on the epigenetic pathways during early development in zebrafish.

Many OPFRs are regarded as endocrine disruptors, acting over many physiological pathways such as lipid metabolism, immunological processes, reproductive system, or steroidogenesis [48,56]. In this regard, we propose, for OPFRs assessment, the inclusion of CE as a generalist marker, given its role in lipid metabolism and metabolic disorders [57,58].

## 5. Conclusions and Limitations

The present study provides a set of novel data of two aryl-OPFRs, TPP and TCP, during early-life of zebrafish. Survival, hatching rate, teratogenic effects, gene expression for detoxification, immune responses, methylation markers, and enzymatic activities (CE and AChE) were evaluated in 5 dpf larvae. Our study highlights the need for further research on OPFRs as sustained exposure during critical developmental windows can lead to disruptive consequences in fish. Although the concentrations assayed were not environmentally relevant, the significance of the data obtained provides grounds for further research.

A larger sample size would have been beneficial to validate the above-mentioned parameters as markers of aryl-OPFR exposures; nonetheless, the outlined expression changes encourage further consideration. Additional experiments involving other enzymatic measurements after aryl-OPFRs exposures, together with (epi)genomic studies, would undoubtedly benefit the discussion and assist to decipher the toxicity mechanisms by which TPP and TCP contribute not only to CEs inhibition but also to the other multiple pathways in which CEs participate.

## Figures and Tables

**Table 1:** Primer sequences information for RT-qPCR experiments.

Gene Name	Gene Symbol	Accession Number	Forward (5′-3′)	Reverse (3′-5′)	Primer Efficiency (%)
Carboxylesterases	*ces2*	NM_001077252.1	GTGGAGCTTGCATGTTTAAGG	GCTGATCTCCTGTGCTGAAGTA	105
DNA (cytosine-5-)-methyltransferase	*dnmt1*	NM_131189	TCTTCAGCACTACAGTTACCAATCCT	CGTGCACATTCCCTGACACT	96
DNA (cytosine-5-)-methyltransferase 3bb.2	*dnmt3*	NM_131386	AAGATTTAGGCGTCGGTTTCG	GTGTCACCCCCTTCAATTAACTG	104
Interleukin 1 β	*il1β*	NM_212844	TGGACTTCGCAGCACAAAATG	GTTCACTTCACGCTCTTGGATG	106
Caspase 9	*casp9*	NM_001007404	CAACATCGACTGCGACAAGC	CAACATCGACTGCGACAAGC	99
Eukaryotic translation elongation factor 1 alpha 1	*eef1a1l1*	NM_131263	CTGGAGGCCAGCTCAAACAT	ATCAAGAAGAGTAGTACCGCTAGCATTAC	95
Ribosomal protein L13a	*rpl13a*	NM_212784	TCTGGAGGACTGTAAGAGGTATGC	AGACGCACAATCTTGAGAGCAG	97

**Table 2:** Percentage of inhibition (%) of enzymatic activities.

Chemical	pNPB-hCE1	ATC-eel AChE
TPP	97.7 ± 0.1	21.6 ± 8.2
TCP	93.8 ± 0.1	58.9 ± 3.3
BNPP	96.6 ± 0.1	NS
BW284c51	NS	99.5 ± 0.1

Human recombinant carboxylesterase (hCE1) and purified eel-acetylcholinesterase (AChE) after in vitro exposure to 100 µM of TPP, TCP, or model inhibitors BNPP (for CE) and BW284c51 (for AChE) in duplicate. Results are expressed in percentage (%). Inhibition over 20% are considered significant [44]. NS = non-significant.

## Data Availability

Data is contained within the article or Supplementary Materials.

## Supplementary Materials

The following supporting information can be downloaded at: https://www.mdpi.com/article/10.3390/toxics13090794/s1, Figure S1: Malformation at 5 days post fertilization (dpf) zebrafish treated with TPP and TCP.
