# Supplementary material for: Modulation of Detoxification, Immune, and Epigenetic Systems by Two Aryl Organophosphorus Flame Retardants During Early Development in Zebrafish
Source: Toxics. 2025 Sep 18;13(9):794. doi: 10.3390/toxics13090794 (PMC12474386; doi:10.3390/toxics13090794)

# Modulation of Detoxification, Immune, and Epigenetic Systems by Two Aryl Organophosphorus Flame Retardants During Early Development in Zebrafish

**Montserrat Solé, Sílvia Joly, Sergi Omedes, Isabel Forner-Piquer and Laia Ribas ***

Institut de Ciències del Mar, Consejo Superior de Investigaciones Científicas (ICM-CSIC), 08003 Barcelona, Spain; msole@icm.csic.es (M.S.); sjoly@icm.csic.es (S.J.); omedes@icm.csic.es (S.O.); iforner@icm.csic.es (I.F.-P.)

* Correspondence: lribas@icm.csic.es

## Supplementary Figure

**Supplementary Figure S1.** Malformation at 5 days post fertilization (dpf) zebrafish treated with TPP and TCP. Four tested families from zebrafish embryos treated with organophosphate flame retardants. Different types of teratologies were observed: (**a**) Control fish, (**b,c**) overall body deformation, and (**d**) tail curvature. Percent of teratologies observed at 5 days post fertilization.

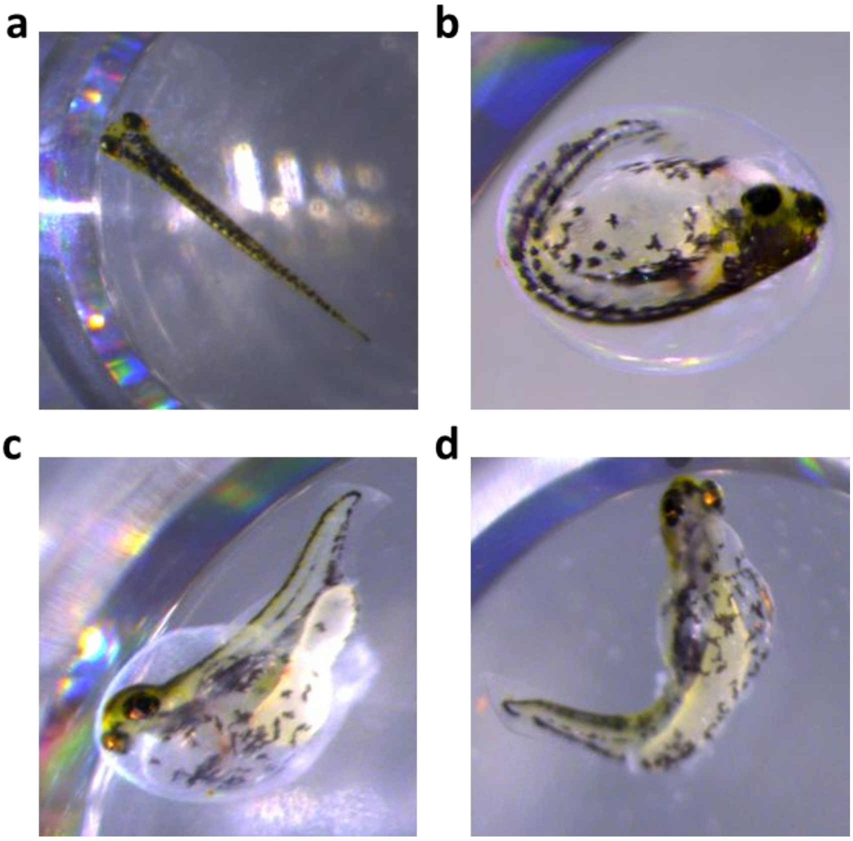

Supplement: Supplementary file 1 [file toxics-13-00794-s001.zip › toxics-3824487-supplementary.pdf]
